# Clusterin and Its Isoforms in Oral Squamous Cell Carcinoma and Their Potential as Biomarkers: A Comprehensive Review

**DOI:** 10.3390/biomedicines11051458

**Published:** 2023-05-16

**Authors:** Qinyi Zhang, Jun Yao Teow, Jesinda Pauline Kerishnan, Adyani Azizah Abd Halim, Yeng Chen

**Affiliations:** 1 Department of Biomedical Science, Faculty of Medicine, Universiti Malaya, Kuala Lumpur 50603, Malaysia; 2 Department of Medicine, Faculty of Medicine, Universiti Malaya, Kuala Lumpur 50603, Malaysia; 3 Department of Oral and Craniofacial Sciences, Faculty of Dentistry, Universiti Malaya, Kuala Lumpur 50603, Malaysia

**Keywords:** oral squamous cell carcinoma (OSCC), oral cancer, clusterin (CLU), therapeutic targets, biomarkers

## Abstract

Oral squamous cell carcinoma (OSCC) is a prevalent type of head and neck cancer, ranked as the sixth most common cancer worldwide, accounting for approximately 300,000 new cases and 145,000 deaths annually. Early detection using biomarkers significantly increases the 5-year survival rate of OSCC by up to 80–90%. Clusterin (CLU), also known as apolipoprotein J, is a sulfated chaperonic glycoprotein expressed in all tissues and human fluids and has been reported to be a potential biomarker of OSCC. CLU has been implicated as playing a vital role in many biological processes such as apoptosis, cell cycle, etc. Abnormal CLU expression has been linked with the development and progression of cancers. Despite the fact that there are many studies that have reported the involvement of CLU and its isoforms in OSCC, the exact roles of CLU and its isoforms in OSCC carcinogenesis have not been fully explored. This article aims to provide a comprehensive review of the current understanding of CLU structure and genetics and its correlation with OSCC tumorigenesis to better understand potential diagnostic and prognostic biomarker development. The relationship between CLU and chemotherapy resistance in cancer will also be discussed to explore the therapeutic application of CLU and its isoforms in OSCC.

## 1. Introduction

Oral squamous cell carcinoma (OSCC) is a commonly diagnosed head and neck cancer characterized by uncontrolled ulcerative lesions in the oral mucosa between the vermilion border of the lips and the oropharynx [1]. It carries a huge global health burden, with approximately 300,000 new cases and 145,000 deaths annually worldwide [2], ranking as the sixth most common cancer globally [3]. The incidence of OSCC varies geographically, with the highest rates found in South and Southeast Asia [4]. The development of OSCC is influenced by a combination of genetic, environmental, and lifestyle factors. The most established risk factors are tobacco smoking, alcohol consumption, and betel quid chewing, with the heavy use of these substances significantly increasing the risk of developing OSCC [5]. Early detection using biomarkers significantly increases the 5-year survival rate of OSCC by up to 80–90% when compared to a survival rate of only 20–40% when diagnosed during the advanced stages [6].

Clusterin (CLU), also known as apolipoprotein J, is a sulfated chaperonic glycoprotein that was first discovered in ram rete testis fluid in 1983 and was observed to enhance cell aggregation in vitro. As a highly conserved glycoprotein, CLU is known to be expressed in all tissues and human fluids and is involved in diverse cellular functions, including cell cycle regulation, apoptotic cell death, DNA repair, membrane recycling, lipid transportation, and immune system regulation [7,8]. Numerous studies have reported the promising potential of CLU as a biomarker for OSCC [9,10]. Despite extensive efforts by thousands of researchers over the last four decades to comprehensively understand this multifaceted protein, its functional properties in various diseases continue to remain an enigma. Recent studies have primarily focused on elucidating its role in lipid metabolism, as well as its involvement in neurodegenerative conditions such as Alzheimer’s disease [11]. In the literature, various transcript variants of CLU have been documented that arise from alternative splicing, with the secretory and nuclear isoforms being the most prevalent. Several studies have reported the involvement of CLU and its isoforms in various cancers, demonstrating their dual role in promoting and suppressing cancer cells [12]. The dysregulated expression of CLU and its correlation with tumorigenesis, along with potential clinical implications, have been extensively documented in multiple types of cancer, including lung cancer, prostate cancer, and breast cancer [13,14,15].

Despite the fact that oral squamous carcinoma cells have been observed to be associated with aberrant CLU expression [10], the specific role of CLU and its isoforms in oral cancer, as well as their potential effects on the susceptibility of cells to tumorigenesis and any clinical implications, have not been fully investigated. Hence, the present review aims to comprehensively discuss the current understanding of CLU and its role in OSCC. In Section 1, we will provide an overview of the structure and molecular functions of CLU, followed by an in-depth discussion concerning the specific role of CLU in OSCC, particularly concerning its isoforms in tumorigenesis and chemoresistance. Finally, we critically appraise and discuss the potential therapeutic application of CLU and its isoforms in OSCC and their use as potential biomarkers for diagnosis and prognosis.

## 2. Clusterin and Its Isoforms

### 2.1. CLU Gene

**Figure 1:**
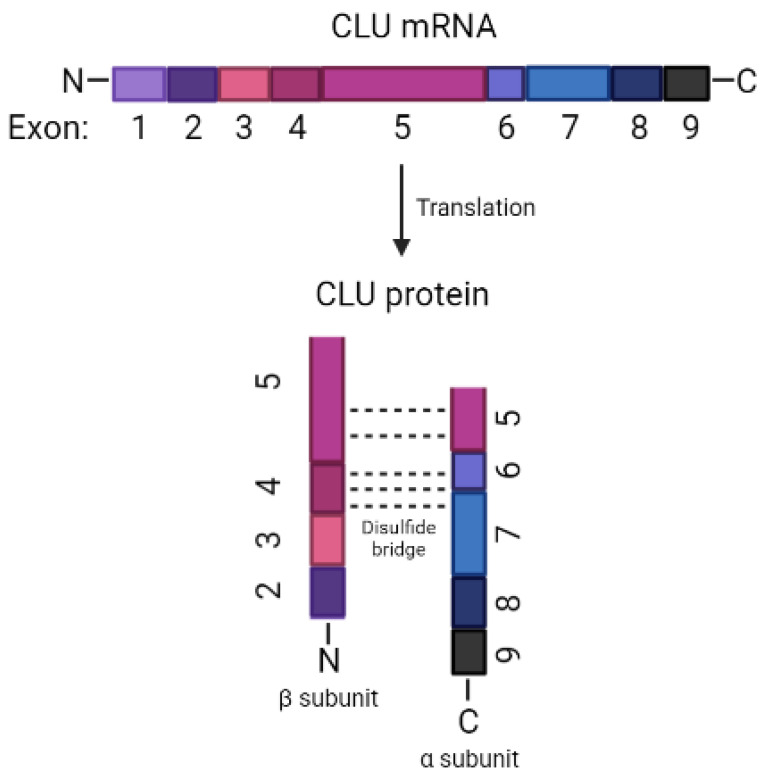
Model of *CLU* mRNA and its translation to CLU protein alpha and beta subunits.

The *CLU* gene is located in human chromosome 8 (8p21-p12), a very well-conserved gene during species evolution [16]. CLU protein is expressed in various physiological fluids, including plasma, semen, urine, breast milk, and cerebrospinal fluid [17]. The *CLU* gene comprises nine exons of different sizes, spanning a region of 17,877 bps to produce three protein forms via alternate splicing with diverse subcellular localizations and biological functions [18]. These nine exons of the *CLU* gene produce a heterodimeric glycoprotein that comprises two disulfide-linked subunits: alpha (34–36 kDa) and beta subunits (36–39 kDa) (Figure 1) [15].

### 2.2. Types of Clusterin Isoforms and Their Structures

All *CLU* isoforms are transcriptions from part of exon 1, but fully from exons 2–9. Exon 1 of the *CLU* gene consists of three portions, namely 1a, 1c, and 1b. With the alternate single portion of exon 1, which contains different terminals in the 5′-untranslated region, isoforms are identified [19]. To date, researchers have tended to report CLU1 and CLU2 as the two main clusterin gene transcripts. In addition, there are also nCLU and sCLU, which are identified in two different locations of the cell. This CLU secretory isoform (sCLU) can only be detected in the cytoplasm. Immediately after the translocation to the Golgi apparatus, the immature sCLU will undergo glycosylation to form a heavily glycosylated complex with carbohydrate moieties. The complex will subsequently be proteolytically cleaved into alpha and beta subunits at an internal site between Arg205 and Ser 206 [20]. Ultimately, the mature sCLU is secreted as a heterodimeric 75–80 kDa glycoprotein complex [21]. Whistle, NuclearCLU (nCLU) is a shorter form of CLU. Under stressful circumstances, it can favor the transcription of a truncated mRNA-CLU lacking exon 2 and the endoplasmic reticulum-targeting peptide. This alteration results in a mature nCLU protein that undergoes alpha/betta cleavage or extensive glycosylation [20].

### 2.3. Regulation of CLU Expression

CLU expression is suggested to be tissue-specific, and the expression level is finely regulated at the transcriptional, translational and post-translational levels [22]. Under normal conditions, CLU expression is well regulated by its basal promoter and regulatory protein binding. With certain healthy or disease statuses, diverse pathways, such as molecular machinery, signaling, or epigenetic modifications, are involved in the control of CLU expression in order to meet necessary changes [23]. In Garcia-Aranda et al.’s findings [20], the articles concluded the possible ways that can lead to the modification of CLU expression. There are four main categories of modification: epigenetic modifications, transcription factors and oncogenes, UTR sequences, and molecule signaling and the cell environment. To further describe them, the epigenetic modification includes histone modification, DNA methylation, and micro-RNAs regulation. Molecule signaling and cell environment involve hormonal and cytokine changes and stress-inducing agents.

### 2.4. Molecular Functions of Clusterin and Its Isoforms

CLU exhibits a dichotomous role in cell apoptosis. This might be due to the different isoforms of CLU that have completely opposite cellular and biological functions in regulating apoptosis [15]. Many studies have been conducted over the years to investigate this dichotomous role of CLU. nCLU (nucleus Clusterin) has been found to be a pro-apoptotic molecule. The C-terminal coiled–coil domain of nCLU formed a complex with Ku70/Ku80 when cancer cells were exposed to ionizing radiation (IR). The complex resulted in decreased cell growth and colony-forming ability due to increased G1 cell cycle arrest and cell death [24]. Kim et al. (2012) [25] also conducted a study that showed the nCLU induced-apoptosis mechanism using Bax as the key molecule. The mechanism involves the ability of nCLU to interact with Bcl-XL, leading to the release of Bax; Bax further activates the release of caspase-3 and cytochrome c, which help promote apoptosis. 

On the other hand, unlike nCLU, a pro-apoptotic molecule, sCLU is an intracellular CLU, which, when secreted into the serum, shows a totally opposite function in terms of regulating apoptosis (an anti-apoptotic molecule). sCLU suppresses Bax activation and relocates it to mitochondria by binding itself to the Ku-70-Bax complex in the cytoplasm, stabilizing it. This helps to prevent the Bax complex from exerting a pro-apoptotic activity at the mitochondrial outer membrane. sCLU also interacts with c-Myc, which promotes the ability of cancer cells to proliferate and progress in vivo [26]. 

sCLU is also found to be associated with the PI3K/AKT axis. High levels of sCLU upregulate the low-density lipoprotein-related protein 2 (LRP-2) known as megalin (which is involved in the endocytic process). Increased megalin receptors lead to phosphorylation and the activation of AKT. Activated AKT leads to the further phosphorylation of the downstream molecule, Bad, which causes a decrease in cytochrome c release. This mechanism helps protect cancer cells from inducing apoptosis due to tumor necrosis factor-α (TNF-α) [27]. 

Another research finding shows that sCLU promotes autophagy through the AMPK/AKT/mTOR signaling pathway when sCLU is overexpressed. This helps to promote cell survival and prevent starvation-induced apoptosis. This is an important factor in maintaining cancer cell survival under nutrient- and oxygen-deficient conditions [28]. Hwang et al. (2014) [29] also demonstrated that the overexpression of sCLU is associated with the angiogenesis of cancer via VEGF signaling. 

Apart from that, sCLU has also been found to regulate extracellular matrix (ECM) remodeling. sCLU complexes with eukaryotic translation initiation factor 3 subunit I (EIF3I) activate AKT signaling; this helps to promote matrix metalloproteinase-13 (MMP-13) expression [30]. Under normal physiological conditions, MMP-13 is usually well-regulated in a relatively low amount. The overexpression and secretion of sCLU are directly proportional to MMP-13 production, which leads to cancer cell metastasis [30,31]. Shim et al. (2011) [32] also hypothesize that matrix metalloproteinase-9 (MMP-9) activity is promoted by sCLU through the activation of the ERK1/2 and PI3K pathways independently of each other. A later study discovered that sCLU promotes the metalloproteinase-2 (MMP-2) matrix through the PI3K/Akt pathway to induce tumor invasion and migration in hepatocellular carcinoma [33].

The physiological functions of sCLU, as shown by the studies mentioned above, are as follows: (i) stabilizing the Ku-70-Bax complex and suppressing Bax activation; (ii) promoting cancer cell survival via PI3K/AKT signaling; (iii) promoting autophagy and the angiogenesis of cancer; and (iv) enhancing cancer metastasis by promoting metalloproteinase expression. In general, elevated sCLU levels are associated with enhanced tumorigenesis as the sCLU is a stress-induced and ATP-independent extracellular chaperone protein. However, it is uncertain whether the overexpression of sCLU in cancer progression is the cause or the consequence of the disease [34].

The presence of two isoforms could account for why the transition to high-grade or metastatic carcinoma involves a switch from nCLU to sCLU expression, and it is possible that the impact of CLU on tumor growth is connected to a change in the production pattern of its isoforms [16].

## 3. Clusterin and Its Isoforms in OSCC

### 3.1. Clusterin and Its Isoforms in Tumorigenesis

CLU expression has been studied, and it has been found to be linked to tumorigenesis in various types of cancer, such as breast, ovarian, liver, and lung cancer [35,36,37]. The overexpression of CLU has been observed during the early stages of tumorigenesis and is associated with the loss of function of tumor suppressor genes. Higher levels of CLU expression have been linked to increased cell proliferation, migration, invasion, and metastasis [38]. However, an increased level of CLU expression may not be solely responsible for the development of cancer. To date, several findings have established the tumor suppressive function of CLU in cancer cells. Although limited studies have reported decreased CLU expression in tumors, there are still a few reports suggesting a reduction in CLU levels in specific cancers, including prostate cancer [39], neuroblastoma [40], and OSCC [10,12]. A pan-cancer analysis has also demonstrated a significant decrease in CLU expression in various cancerous tumors [41]. In fact, due to the dual role of clusterin as a tumor suppressor and a tumor promoter, currently, it remains uncertain whether cancer specifically expresses only the anti-apoptotic form of CLU or if pro-apoptotic nCLU isoforms are consistently suppressed in different types of cancers.

In a recent investigation conducted by Kadam et al. (2020) [12], sCLU showed a significant correlation with OSCC. The nucleolar localization of sCLU and its link with Cajal bodies were demonstrated in response to post-nucleolar stress, indicating its significance in maintaining nuclear morphology in oral cancer. Additionally, the inhibition of sCLU was found to change nuclear morphology and lead to shrinkage in tubulin filaments, suggesting the tumor-suppressive function of CLU. Furthermore, in the investigated oral cancer cell lines and tumor tissues, Kadam et al. (2020) [12] were unable to detect nCLU transcripts, which tallies with Prochnow et al.’s (2013) [42] findings regarding various cancer and non-cancer cell lines, where sCLU predominates, and nCLU expression levels are low. Notably, Prochnow et al. (2013) [42] discovered an increase in nCLU transcripts following proteotoxic stress, while Kadam et al. (2020) [12] found no alterations in nCLU transcripts following nucleolar stress in oral cancer cell lines, suggesting that sCLU is possibly accumulated in the nucleolus. 

**Figure 2:**
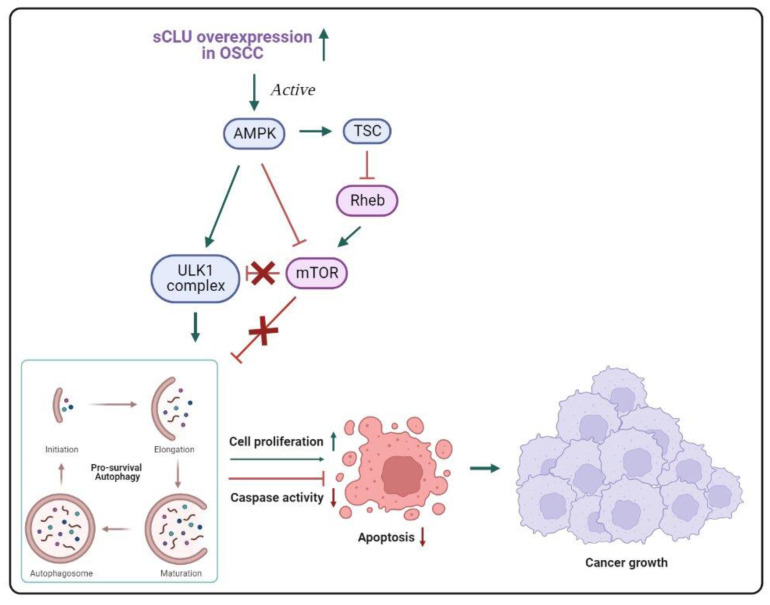
Model of autophagy regulation by the AMPK/mTOR/ULK1 pathway due to overexpression of sCLU in OSCC. AMPK is activated, and mTOR is inhibited in sCLU overexpression; Ulk1 is activated to initiate autophagy to prevent starvation-induced apoptosis.

In comparison with Kadam et al.’s (2020) [12] study, the findings of a study conducted to investigate the effect of sCLU-mediated autophagy on cell survival and apoptosis inhibition in oral cancer revealed a rise in the expression levels of sCLU that correlated with increasing tumor grade in OSCC, suggesting a potential cancer-promoting role of sCLU [28]. In Naik et al. (2021) [28], the anti-apoptotic activity of sCLU is manifested through its regulation of the AMPK/mTOR/ULK1 axis (Figure 2), which results in the initiation of autophagy. Although autophagy has been recognized as a tumor suppression mechanism, studies have revealed its crucial involvement in sustaining the survival of cancer cells, particularly under hypoxic and nutrient-deprived conditions, which are commonly present in the tumor’s central region, ultimately promoting the growth and progression of cancer [43,44,45]. According to Naik et al.’s (2021) [28] findings, the overexpression of sCLU in OSCC results in a substantial upregulation of AMPK expression. AMPK plays a vital role as an energy sensor and helps regulate cellular metabolism through phosphorylation at Thr172. When phosphorylated, phospho-AMPK (Thr172) activates the TSC2 protein, which, in turn, inhibits the downstream targets such as mTOR and S6K, leading to the induction of autophagy. sCLU also interacts with ULK1 to facilitate starvation-induced autophagy. Furthermore, sCLU upregulates mitophagy-associated proteins during serum starvation, suggesting that sCLU promotes autophagy to help protect cells against apoptosis in response to nutrient deprivation. Table 1 summarizes the distinct roles of CLU isoforms in OSCC.

In addition to the molecular mechanisms, tobacco smoking, as a major risk factor for the development of oral cancer, has recently been discovered to be linked with CLU. In contrast, the expression of CLU did not exhibit a correlation with nicotine addiction or dependence scores, and a significant increase in CLU levels was observed in the saliva of individuals who engaged in prolonged tobacco use or high-intensity tobacco consumption. Furthermore, the level of CLU protein was found to be markedly decreased within six months after smoking cessation, suggesting the possible connection between CLU and tobacco smoking [46]. Benzo(a)pyrene (BaP) is an environmental contaminant that is widely distributed and has been identified as a component in mainstream cigarette smoke [47]. It has been suggested that BaP may play a role in the induction of salivary CLU expression during tobacco smoking [15]. A previous study has found an association between CLU and BaP in lung cancers; specifically, the overexpression of CLU was observed in BaP-transformed 16HBE cell line T-16HBE-C1 cells [48]. Interestingly, several studies have reported associations between BaP and OSCC [49,50]. BaP has been shown to regulate the expression of specific genes that are involved in tumor-associated signaling pathways that promote cancerous cell survival by stimulating OSCC cell proliferation and metastasis while simultaneously suppressing apoptosis [50]. Further research is necessary to explore whether there exist mechanisms linking CLU and BaP or other components in tobacco to the development of OSCC.

### 3.2. Impact of Clusterin on Resistance to Chemotherapy

During the last two decades, there has been an increasing inclination toward using a combination of chemotherapy, radiation therapy, and surgery to treat patients suffering from advanced, recurrent, or metastatic head and neck cancer [51]. Various studies have revealed the critical role of sCLU in stimulating resistance to chemotherapy in a range of cancers [52,53,54]. Cisplatin is a platinum-containing compound frequently used in combination with fluorouracil (5-FU) and docetaxel for the treatment of oral cancer. Research carried out using OSCC cell lines suggests that autophagy mediated by sCLU may contribute to chemoresistance through the inhibition of apoptosis in oral cancer [28]. In Naik et al.’s study, it was found that the expression of a critical protein in controlling autophagy, ATG14, displays a linear response in OSCC in terms of the progression of cancer [28]. A similar finding was shown in another study concerning ovarian cancer, which reported that inhibiting cytoprotective autophagy by reducing ATG14 expression promotes cisplatin-induced apoptosis and increases sensitivity to chemotherapy [55]. In a recent investigation on oral cancer stem cells, it was discovered that treating the cells with cisplatin promoted the activation of mitophagy—a mechanism for the elimination and prevention of damaged mitochondria accumulation to avoid cell death—by regulating the levels of CLU. Remarkably, when the researchers reduced CLU expressions, it disrupted the mitochondrial metabolism of the cells, leading to the accumulation of deleterious mitochondrial superoxide, thereby augmenting the sensitivity of the cancer cells to cisplatin treatment [56]. 

Although there is a lack of research on the relationship between CLU and docetaxel or 5-Fu resistance in OSCC, studies conducted on other types of cancer have demonstrated a correlation that could potentially be relevant to oral cancer. In 2010, Zhong et al. [57] shed light on the role of AKT and sCLU in tumor cell survival and chemoresistance in prostate cancer. The study found that AKT is responsible for inducing sCLU through the activation of the signal transducer and activator of transcription 1, leading to an overexpression of sCLU, which subsequently resulted in resistance to docetaxel [57]. In 2020, a different mechanism was reported in gastric cancer, where an increase in the expression of sCLU led to reduced sensitivity to docetaxel through the suppression of miR-195-5p [52]. Due to the fact that many studies have demonstrated that suppressing - CLU activity could enhance the efficacy of chemotherapeutic agents in eradicating cancerous cells, the downregulation or targeted silencing of the *CLU* gene may provide a promising future in terms of therapeutic interventions for oral cancer.

## 4. Potential Clinical Applications of Clusterin Isoforms in OSCC

### 4.1. Use of Clusterin Isoforms as Diagnostic and Prognostic Biomarkers

In fact, many studies have reported findings related to the association between CLU expression and cancer incidence and progression, such as in lung cancer, prostate cancer, colon cancer, and hepatocellular carcinoma [13,58,59,60]. It is not a surprise that CLU might also be a good candidate as an OSCC biomarker. However, the expression of sCLU has been observed to vary across a wide range of malignancies. OS prognosis study data from Fu et al. (2023) [41] suggest varied conclusions regarding different types of cancer, *CLU* gene expression, which leads to a worse prognosis, is inconsistent across different cancers. The question that remains here concerns the uncertainty as to whether sCLU, which is anti-apoptotic, is the only type of CLU expressed in cancer or if nCLU is suppressed in various tumor types. 

According to a study conducted by Fu et al. (2023) [41], CLU expression has been demonstrated to be decreased in most cancer types. One phenomenon observed in the prognosis of most cancer patients is that the CLU gene is always found to have a significant deletion in all malignancies. In fact, CLU has been reported to exhibit tumor suppressor activity in lung and oral cancers [12,61]. Worse prognosis and genetic instability are accompanied by low CLU expression. Interestingly, a totally reverse scenario is seen in kidney renal clear cell carcinoma (KIRC) and liver hepatocellular carcinoma, where CLU expression is correlated with worse cancer survival [62,63]. This discrepancy is believed to be caused by CLU isoforms, sCLU, which has a negative effect on cancer prognosis [41]. sCLU is involved in many mechanisms mentioned above, which ultimately contribute to anti-apoptotic properties. A clear example demonstrated by Wang et al. (2018) [64] shows the ability of sCLU to inhibit mitochondria apoptosis in hepatocellular carcinoma by activating the PI3K/Akt pathway through the suppression of Gadd45a expression, a negative regulator of the PI3K/Akt pathway. Yao et al. (2018) [65], Ma et al. (2019) [66], and Chen et al. (2021) [67] also proposed the potential use of sCLU as a biomarker in liver cancer, osteosarcoma, and breast cancer, respectively. 

Many findings produced through research concerning OSCC concluded the significant downregulation of CLU in patient serum and salivary samples compared to normal individuals [9,68,69]. The results suggested that *CLU* could act as a suppressor of tumors during the initial phase of cancer development. In advanced stages of cancer, reduced levels of CLU could activate nuclear factor-κB (NF-κB), which is associated with inflammation, leading to the increased proliferation and invasion of cancer cells. [70]. However, there are also studies that found the overexpression of sCLU in OSCC in a grade-wise manner [28,38]. These discrepancies may arise from the fact that different CLU isoforms are being expressed. It is known that CLU functions in an isoform-dependent manner, and CLU isoforms exhibit both pro-apoptotic and anti-apoptotic properties [39]. Praharaj et al. (2021) [38] proposed promoted OSCC cell survival and the prevention of induced apoptosis caused by prolonged exposure to serum starvation due to the overexpression of sCLU toward the AMPK/Akt/mTOR pathway. Notably, the relationship between CLU and OSCC remains elusive as the true function of CLU in relation to OSCC progression is undefined.

The downregulation of CLU has also been addressed in another study that investigated the sCLU expression in prostate carcinoma cells [39]. Interestingly, sCLU expression seems to be area-specific as it is undetectable in carcinoma cells but present in stromal peritumoral fibroblasts. Scaltriti et al. (2004) [39] also reported sCLU accumulation in the different specific areas of tumors during tumor stage progression.

Another perspective to look at concerning CLU protein association with cancer is the level of CLU methylation. CLU methylation has been found to be at a significantly higher level in prostate adenocarcinoma (PRAD) compared to non-tumor tissues. Future study is needed as the effects and consequences of CLU methylation in cancer tumor initiation and progression are currently unknown [41].

The CLU expression levels in OSCC seem to be inconsistent and there is a need for an improved plan to elucidate the eligibility of CLU as a diagnostic and prognostic biomarker of OSCC. Chen et al. (2014) [9] provided another insight by investigating the immunogenicity of the proteins concerning their expression levels. Immunogenic CLU can only be detected in the serum of OSCC patients. This immunogenicity property could be due to the post-translational modification (PTM) or insertion of certain foreign or unknown fragments into the CLU. Therefore, studies concerning sCLU versus nCLU in terms of gene expression levels and structural modification at the protein level could be good alternative ideas to further evaluate the association of CLU with OSCC progression [8]. The potential application of CLU isoforms is summarized in Table 2.

### 4.2. Use of Clusterin Isoforms in Personalized Treatment Strategies

A recent study concluded that sCLU is overexpressed and increases in OSCC tumors in a grade-wise manner, implying its pro-cancer role in OSCC [28]. Zhang et al. (2016) [71] conducted a meta-analysis that also supports the crucial role played by sCLU in cancer progression. The primary function of sCLU is believed to boost the expression of cancer cell survival proteins to aid tumor progression [72]. Thus, sCLU may be a promising therapeutic target in terms of personalized treatments for OSCC. 

However, sCLU targeting is challenging as CLU undergoes post-translational modifications making it hard to inhibit through the use of small molecule inhibitors [38]. In that sense, siRNA-based genetic inhibition or antisense-based inhibition are the suggested options to suppress sCLU expression in cancer. Several studies have been conducted to investigate the effects of sCLU suppression on cancer treatment modalities. A study conducted by Ma et al. (2019) [66] found that sCLU silencing would help reduce the oncogenic and metastatic potential of osteosarcoma (OS) cells, OS cells’ growth and survival rate are depleted. Silenced sCLU also increases the OS cells’ sensitivity towards chemotherapeutic drugs as the sCLU increases the OS cells’ chemoresistance; sCLU also inhibits Bax-dependent apoptosis by stabilizing the Bax-Ku70 complex [73]. Ma et al. (2019) [66] suggested sCLU targeting for cancer treatment. Another study proposed that the lentiviral transduction of shRNA can be used to knock down sCLU expression in ovarian cancer to promote cell cycle arrest to prevent cancer proliferation, migration, and invasion [74]. A study conducted recently revealed that knocking down the *CLU* gene would lead to an imbalance in the mitochondrial metabolism, causing an excess of a reactive oxygen molecule called mitochondrial superoxide. This imbalance, in turn, increased the sensitivity of oral cancer stem cells (CSCs) to cisplatin treatment which is a chemotherapeutic agent [56]. Interestingly, the research also showed that the cytoprotection effect of CLU is dependent on the expression of SOX2. When SOX2 expression was reduced using genetic (shSOX2) or pharmacological (KRX-0401) methods, the protective effect of CLU was reversed, leading to increased sensitivity of oral CSCs to cisplatin-induced cell death.

In addition to using a genetic inhibition method, the antisense oligonucleotide-based approach is also utilized in clinical stages to treat different types of cancer. Custirsen (OGX011) is an antisense oligonucleotide (ASO) that is used to target the ATG sequence present on the sCLU exon 2. The ATG sequence is the initiation codon, and the formation of a DNA-RNA duplex would inhibit the translation of sCLU [75]. The use of OGX-011 and zoledronic acid (ZOL) together in combination therapy effectively stopped the growth of tumors and prolonged tumor survival in osteosarcoma. This was achieved by reducing the transcription of several regulatory factors, including CLU, MDR1, and Heat shock factor 1 [38]. Another study concerning Caki-2 cells (human clear cell carcinoma of the kidney) showed that CLU ASO inhibits *CLU* mRNA expression. Although CLU ASO alone did not have any effect on the Caki-2 cells’ growth, increased apoptosis was seen when CLU ASO was used in combination with cytotoxic chemotherapy [76].

July et al. (2004) [77] conducted a study on nucleotide-based therapies targeting sCLU (either ASO or siRNA) in human lung adenocarcinoma cells in vitro and in vivo, resulting in increased chemosensitivity to drugs. The in vivo application of sCLU ASO significantly improved the impact of paclitaxel or gemcitabine in delaying the growth of A549 tumors in immunocompromised mice in a synergistic manner. 

Inhibitors against the upstream or downstream of the CLU regulator could also be an alternative plan for CLU-based treatment process. For instance, small molecule inhibitors target IGF-1R, a protein that controls the IGF-1R survival pathway, which also leads to reduced sCLU [78]. Ascorbate has also been found to reduce anti-apoptotic cytoplasmic CLU at both the transcriptional and protein levels without interfering with pro-apoptotic nCLU that triggers cell death in melanoma cells [79]. Several miRNAs, including has-miR-17-5p, has-miR-21-5p, has-miR-425-5p, and has-miR-18a-3p, have been identified to have inhibitory effects on CLU, supported by experimental evidence [80]. Research focusing on more potent miRNAs would be a good strategy for contributing to CLU inhibition-based cancer therapy. The combination of FDA-approved chemotherapeutic drugs with CLU inhibitors is demonstrated by these findings to be effective in treating different types of cancer, leading to improved therapeutic outcomes.

## 5. Conclusions

In conclusion, this review highlights the crucial role of Clusterin in Oral Squamous Cell Carcinoma, specifically its isoforms, in the development and progression of OSCC. Despite the fact that a large amount of evidence has suggested the potential of the use of CLU as a biomarker, focusing solely on its aberrant expression may not be sufficient. The lack of research concerning CLU isoforms in OSCC calls for more evidence to establish correlations and explore their diagnostic or prognostic potential as well as therapeutic targets for effective cancer therapy. Future research is required to address the challenges of clarifying the specific roles of different CLU isoforms and exploring the detailed molecular mechanisms to enhance our understanding of CLU’s role in OSCC carcinogenesis. Targeting CLU and its isoforms is believed to provide new avenues for discovering novel therapies and enhancing patient outcomes.

## Figures and Tables

**Table 1:** Roles of CLU isoforms in OSCC.

CLU Isoforms	Regulation	Specific Roles	Reference
nCLU	Undetectable	Unknown	[12]
sCLU	Down	Tumor suppression; Associated with Cajal bodies in maintaining nuclear morphology	[12]
	Up	Initiates autophagy through regulation of the AMPK/mTOR/ULK1 axis; Interacts with ULK1 to facilitate starvation-induced autophagy; Upregulates mitophagy-associated proteins during serum starvation	[28]

**Table 2:** Potential Clinical Applications of Clusterin Isoforms In OSCC.

Potential Application	Description	Reference
sCLU versus nCLU level	Association of sCLU or nCLU towards OSCC progression.	[8]
Immunogenicity of CLU	Immunogenicity properties of CLU are detected in OSCC patient serum due to PTM or other unknown fragments.	[9]
CLU expression level	CLU expression decreases in most cancer types, including OSCC.	[12]
	CLU suppresses tumors in initial OSCC phase, downregulated CLU expression is associated with the increased inflammation, proliferation, and invasion of cancer cells.	[70]
sCLU expression level	sCLU contributes to the anti-apoptotic properties of OSCC. sCLU overexpression promotes OSCC cell survival and prevents starvation-induced apoptosis.	[38]
CLU methylation	High CLU methylation seen in prostate adenocarcinoma (PRAD). Future study is needed to investigate to the relationship between CLU methylation and cancer progression.	[41]

## Data Availability

Not applicable.
